# SENSE OF PURPOSE AMONG OLDER ADULT VOLUNTEERS DURING THE COVID-19 PANDEMIC

**DOI:** 10.1093/geroni/igad104.2672

**Published:** 2023-12-21

**Authors:** Lindsay Wilkinson, Mollie George, Julie Masters, Julie Blaskewicz Boron

**Affiliations:** University of Nebraska Omaha, Omaha, Nebraska, United States; University of Nebraska Omaha, Omaha, Nebraska, United States; University of Nebraska Omaha, Omaha, Nebraska, United States; UNO, Omaha, Nebraska, United States

## Abstract

The psychological benefits of volunteering among older adults are well established, but little is known about the influence of the pandemic on this relationship. Indeed, the COVID-19 pandemic led to social distancing, closures, and other restrictions that hampered the volunteer efforts of many older adults. Utilizing data from a survey of older adult volunteers participating in Foster Grandparent and Senior Companion Programs in a metropolitan area of Nebraska, this study investigates sense of purpose during the COVID-19 pandemic. The analytic sample was comprised of 65 adults ages 57 to 89 years. Linear regression models adjusting for demographic characteristics were used to examine the association between the perceived impact of the pandemic and disruptions to volunteering, and whether disruptions to volunteering were subsequently linked to a reduced sense of purpose. The results reveal that 1 in 4 respondents somewhat or strongly agreed that their sense of purpose had declined during the pandemic. Compared to older adults who perceived that the pandemic had little effect on them, those who perceived that the pandemic had been either difficult or a hardship more strongly agreed that it had been challenging to visit with their clients (both ps < 0.05). Older adults who agreed that there were new challenges in their volunteer role, in turn, more strongly agreed that their sense of purpose had declined (p < 0.01). These findings highlight the importance of volunteering for sense of purpose during the pandemic and suggest a possible pathway through which the pandemic may influence future health.

